# Depressive symptoms partially mediate the relationship between psychosocial factors and epigenetic age acceleration in a multi-racial/ethnic sample of older adults

**DOI:** 10.1016/j.bbih.2025.100994

**Published:** 2025-04-12

**Authors:** Lauren A. Opsasnick, Wei Zhao, Lauren L. Schmitz, Scott M. Ratliff, Jessica D. Faul, Xiang Zhou, Belinda L. Needham, Jennifer A. Smith

**Affiliations:** a Department of Epidemiology, School of Public Health, University of Michigan, Ann Arbor, MI, United States of America; b Survey Research Center, Institute for Social Research, University of Michigan, Ann Arbor, MI, United States of America; c Robert M. La Follette School of Public Affairs, University of Wisconsin-Madison, Madison, WI, United States of America; d Department of Biostatistics, School of Public Health, University of Michigan, Ann Arbor, MI, United States of America; e Division of General Internal Medicine, Northwestern University Feinberg School of Medicine, Chicago, IL, 60611, United States of America

**Keywords:** Social epigenomics, Psychosocial stress, Loneliness, Depressive symptoms, Epigenetic aging, Epigenetic clocks

## Abstract

Psychosocial factors, including cumulative psychosocial stress and loneliness, have been linked to epigenetic aging in older adults. Further, depressive symptoms have established relationships with both psychosocial factors and epigenetic aging. However, it is not known whether depressive symptoms mediate the association between psychosocial factors and epigenetic aging.

We conducted linear regression models to examine associations between psychosocial stress, loneliness, and depressive symptoms and five epigenetic age acceleration (AA) measures estimated by DNA methylation in a multi-racial/ethnic sample of 2681 older adults from the Health and Retirement Study (mean age: 70.4 years). For all identified associations, we tested for effect modification by sex and educational attainment and performed mediation analysis to characterize the role of depressive symptoms on these associations.

Psychosocial stress, loneliness, and depressive symptoms were each associated with at least one measure of epigenetic AA (FDR q < 0.05). Further, we observed interactions between loneliness, psychosocial stress, and sex on DunedinPACE, as well as loneliness and educational attainment on GrimAA, PhenoAA, and DunedinPACE, with females and individuals without a college degree appearing more sensitive to the psychosocial effects on epigenetic aging. Depressive symptoms mediated between 24 % and 35 % of the relationships between psychosocial stress and HannumAA, GrimAA, and DunedinPACE, as well as 40 % and 37 % of the relationships between loneliness and both GrimAA and DunedinPACE, respectively.

**Results:**

from this study may help elucidate the relationship between psychosocial factors and epigenetic aging, which is critical in understanding the biological mechanisms through which psychosocial factors may contribute to age-related disease.

## 1 Introduction

Psychosocial factors, defined as social, cultural, or environmental phenomena that impact an individual's thoughts or behaviors (Psychology), have been associated with a range of mental and physical health outcomes, including age-related diseases (Willis et al., 1997; Everson-Rose and Lewis, 2005). One such factor is psychosocial stress, broadly defined as the perception of threat that results in discomfort and emotional tension (Buscemi et al., 2017). Psychosocial stress has well-established relationships with cardiometabolic disease and its corresponding risk factors, including hypertension and diabetes (Santosa et al., 2021; Spruill et al., 2019; Silverstein-Metzler et al., 2022), as well as autoimmune disorders (Song et al., 2018; Kemeny and Schedlowski, 2007). Similarly, loneliness, which is a state of distress that arises from the perception that one's social needs are not being met by their relationships (Hawkley, 2022), is associated with an increased risk of cardiovascular disease (Albasheer et al., 2024; Golaszewski et al., 2022), functional disability (Qi et al., 2023), cognitive decline, and dementia (Salinas et al., 2022; Huang et al., 2023).

More recently, evidence has linked psychosocial factors to biological aging, defined as the amount of damage that has occurred to the body's cellular functions, including changes to the epigenome (Li et al., 2023a). Epigenetic mechanisms, such as DNA methylation, are biochemical modifications to DNA that alter gene activity without changing the underlying genetic sequence (Berger et al., 2009). Epigenetic clocks are measures of biological aging developed from DNA methylation patterns across the genome (Duan et al., 2022). First-generation clocks, which include HannumAge (Horvath, 2013) and HorvathAge (Hannum et al., 2013), were designed to predict chronological age. However, because they were trained exclusively on age, they may not have selected the most relevant DNA methylation sites, as evidenced by their relatively weak associations with measures of morbidity and mortality (Vasileva et al., 2023). Second-generation clocks, such as GrimAge (Lu et al., 2019) and PhenoAge (Levine et al., 2018), were trained on a combination of health biomarkers or outcomes and designed to predict age-related disease status and mortality. Finally, third-generation clocks, including DunedinPACE (Belsky et al., 2022), were developed using longitudinal change of age-related biomarkers to assess physiological decline in a cohort of individuals with the same chronological age. HorvathAge, HannumAge, PhenoAge, and GrimAge all measure biological age in years, while DunedinPACE measures the pace of aging, such that each unit represents an average rate of one year of biological aging per year of chronological aging. Because each set of epigenetic clocks were trained on different outcomes and markers, differences in the associations between individual clocks and sociodemographic characteristics have been observed within the same study population (Watkins et al., 2023). Thus, when studying epigenetic aging, it is important to consider multiple clocks to better understand the biological impact of specific exposures on each epigenetic aging measure.

Epigenetic clocks are often used to capture epigenetic age acceleration (EAA), defined as “the phenomenon in which an individual's epigenetic age is greater than expected based on his or her chronologic age (Uchehara et al., 2023).” Though epigenetic age and chronological age are correlated, epigenetic age is often more predictive of an individual's physical and mental health than chronological age (Ho et al., 2023). Thus, identifying factors that drive differences between chronological age and epigenetic age, including sociodemographic and psychosocial factors, is crucial to understanding the pathology of aging.

Previous studies have identified associations between measures of psychosocial stress and EAA measured by several clocks (Zannas et al., 2015; Skinner et al., 2024; Harvanek et al., 2021). Yet, results have been somewhat inconsistent, with other studies reporting no significant relationships (Vetter et al., 2022; Zannas, 2019). In a systematic review of the impact of psychosocial stress on epigenetic aging, two studies found positive associations between lifetime stress and epigenetic aging measured by GrimAge and HorvathAge, while the remaining four studies did not observe any significant associations (Lim et al., 2022). Recent studies have also discovered associations between loneliness and accelerated epigenetic aging, measured by GrimAge and DunedinPACE (Freilich et al., 2024; Beach et al., 2022). However, these studies were only conducted in participants during midlife. Because the epigenome can change substantially during the lifespan, it is not clear whether these findings would persist in a population of older adults.

Furthermore, the pathway linking these psychosocial factors to epigenetic aging is not fully understood. One possible mechanism that may elucidate the relationship between psychosocial factors and epigenetic aging is mental health outcomes, including depression and its symptoms (Beydoun et al., 2022). There are well-established relationships between psychosocial factors and depressive symptoms, with studies finding that an increase in both psychosocial stress and loneliness is associated with elevated depressive symptoms (Mushtaq et al., 2014; McGonagle and Kessler, 1990). Additionally, extensive research has shown associations between depressive symptoms and accelerated epigenetic aging measured by multiple clocks (Wang et al., 2023; Liu et al., 2022). Thus, depressive symptoms may mediate the relationship between psychosocial factors and epigenetic aging.

In this study, we examined associations between psychosocial stress, loneliness, and depressive symptoms and five measures of epigenetic age acceleration (EAA) in a multi-racial/ethnic sample of older adults. Additionally, we tested for effect modification by sex and educational attainment to assess sociodemographic differences in the effects of psychosocial factors and depressive symptoms on epigenetic aging. Finally, we performed mediation analysis to understand the role of depressive symptoms in the associations between psychosocial factors and epigenetic aging. Results from this study may help to characterize the relationship between psychosocial factors and epigenetic aging, which is crucial in understanding the biological mechanisms through which psychosocial factors may contribute to age-related disease such as cardiometabolic conditions (e.g., coronary heart disease, diabetes, hypertension, obesity), cancer, and autoimmune disorders (Osborne et al., 2020).

## 2 Materials and methods

### 2.1 Sample

The Health and Retirement Study (HRS) is a longitudinal panel study of U.S. individuals over age 50 and their spouses/partners. 10.13039/100005859HRS, which began in 1992, consists of biennial surveys covering topics such as income and wealth, physical and mental health, cognition, work, retirement, and family support (Juster and Suzman, 1995; Sonnega et al., 2014). Beginning in 2006, a random 50 % of participants were administered the Psychosocial and Lifestyle Questionnaire, or Leave Behind Questionnaire (LBQ), which is a self-completed survey capturing information on participants’ psychological well-being, lifestyle, and social relationships (Crosswell et al., 2020). The remaining half of the sample was administered the LBQ in 2008, and data from this survey is available for all participants longitudinally every four years. Furthermore, in 2016, all panel participants who completed the HRS survey were invited to participate in an ancillary study, the Venous Blood Study (VBS, n = 9934), where blood-based biomarkers were collected (Crimmins et al., 2016). A random subsample of participants who completed the VBS blood draw had their DNA methylation measured (n = 4104). This analysis included 2681 participants who had complete data on psychosocial factors, sociodemographic covariates, and DNA methylation measured from the VBS blood draw.

To evaluate the impact of restricting the analysis to those with complete data, we compared sociodemographic characteristics and health behaviors between those who were included (n = 2681) and excluded (n = 1337) in the primary analysis from the total DNA methylation sample, using student's t-tests and Chi-squared tests, as appropriate. Further, we calculated Cohen's d and Cramer's V effect sizes for continuous and categorical variables, respectively, to quantify the size of the differences between the two groups.

### 2.2 DNA methylation

DNA methylation was measured from whole blood in a sample of racially and socioeconomically diverse HRS participants using the Illumina Infinium HumanMethylationEPIC Beadchip v.1.0 (HRS Epigenetic Clocks – Release 1, 2020). DNA samples were randomized across plates by demographic variables, including age, sex, cohort, education, and race/ethnicity. Data processing and quality control were performed using the *minfi* R package (Aryee et al., 2014). Approximately 3.4 % of methylation probes (n = 29,431 out of 866,091) were removed because the detection p-value fell below a threshold of 0.01. Further, sex mismatched samples and any controls (cell lines, blinded duplicates) were removed prior to analysis. Methylation data was available for 97.9 % of participants (n = 4018) who passed quality control.

### 2.3 Epigenetic clocks and age acceleration

Five epigenetic clocks estimated from the HRS methylation data were examined as outcomes in this study (HRS Epigenetic Clocks – Release 1, 2020). HorvathAge, developed in samples of 51 healthy tissues and cell types, was defined based on 353 CpGs sites, while HannumAge was developed from whole blood samples using 71 CpG sites. PhenoAge, which included 513 CpGs sites, was developed to estimate mortality risk using nine aging biomarkers in whole blood samples. GrimAge, which was developed to capture seven methylation-based surrogate markers of plasma protein and smoking-pack years in whole blood samples, included 1030 unique CpG sites. Finally, DunedinPACE, which measured within-individual decline across 19 biological markers in whole blood samples, included 173 CpG sites. All clocks are publicly available (see https://hrsdata.isr.umich.edu/data-products/epigenetic-clocks), aside from DunedinPACE, which was created using the DunedinPACE algorithm in Belsky et al. (2022). For all clocks except DunedinPACE, we first regressed an individual's epigenetic age on their chronological age and chronological age squared and then extracted the residual values to represent EAA for each clock. EAA quantifies the degree to which an individual's age is accelerated compared to their chronological age. Thus, a positive residual value, or EAA measure, indicates that an individual's epigenetic age is greater than their chronological age, signifying faster aging. Both age and age squared terms were included in the models to account for potential non-linear relationships between age and the epigenetic clocks (Krieger et al., 2023). Because both age and sex are used in the GrimAge calculation, sex is also regressed out of the measure for GrimAge (Faul et al., 2023). DunedinPACE, which is distinct from the other clocks, represents years of accelerated aging per chronological year, with values larger than zero indicating a faster pace of aging (Belsky et al., 2022).

### 2.4 Psychosocial factors

**Cumulative Psychosocial Stress** Psychosocial stress was calculated using data from the 2010 and 2012 Psychosocial Leave Behind Questionnaires (Crosswell et al., 2020). To investigate the impact of lifetime stress exposure on epigenetic aging, we created a cumulative score that captured both acute and chronic stressors that spanned the life course. Six domains of psychosocial stress were included in the cumulative stress score, including: 1) acute life events, 2) financial stress, 3) neighborhood stress, 4) relationship stress, 5) lifetime discrimination, and 6) childhood adversity (Opsasnick et al., 2024a, Opsasnick et al., 2024b). Each domain was made up of one or more stress measures (see Supplemental Table 1, from Opsasnick et al. (2024a)). Details regarding the individual stress measures, as well as the construction of the cumulative stress score, can be found elsewhere (Opsasnick et al., 2024a, Opsasnick et al., 2024b; Cuevas et al., 2019, Cuevas et al., 2021). In summary, each stress measure was transformed into a z-score, and z-scores from the individual stress measures within each domain were summed and re-standardized to obtain domain specific standardized scores. Finally, the standardized scores from each of the six domains were summed and subsequently standardized to calculate the cumulative psychosocial stress score.

**Loneliness** was captured in either 2010 or 2012 using the 11-item Revised UCLA Loneliness Scale (R-UCLA) from the Psychosocial Leave Behind Questionnaire (Russell, 1996; Lee and Cagle, 2017). The full list of items can be found in Supplemental Table 2. Participants were asked to respond (1 = often, 2 = some of the time, 3 = hardly ever or never) to each of the items, and the scores from each item were averaged to create a loneliness index. To allow for comparisons between psychosocial factors, we then standardized the index to create a loneliness z-score.

### 2.5 Depressive symptoms

Depressive symptoms were assessed in 2014 using the modified 8-item Center for Epidemiological Studies Depression (CES-D) scale (Radloff, 1977), which can be found in Supplemental Table 3. Participants were asked to respond (yes/no) to having experienced 8 depressive symptoms over the past week. The individual responses were summed for a final score ranging from 0 to 8, with a higher score indicating a greater number of depressive symptoms. Subsequently, participants’ scores were standardized to create depressive symptoms z-scores. For participants who did not complete the CES-D scale in 2014, their 2016 scores were instead used.

### 2.6 Covariates

Sociodemographic factors and health behaviors were collected during the participant's baseline interview, as well as their 2010 or 2012 HRS core interview, in parallel with the Psychosocial Leave Behind Questionnaire. The following sociodemographic variables were included as covariates: sex (male or female), educational attainment (no degree, high school degree, or college degree or higher), marital status (married/partnered, single, or widowed/divorced), having children (yes/no), employment status (yes/no), and total household wealth in US dollars. The top 10 principal components (PCs) of genetic ancestry, which were estimated using genotype data, accounted for population stratification and admixture (Health and Retirement Study, 2021). Furthermore, the Housman method was used to adjust for white blood cell (WBC) proportions (Houseman et al., 2012).

Health behaviors, including smoking status (never, former, current), alcohol use (never, moderate, heavy), and BMI (normal, overweight, obese class I, obese class II) were additional covariates included in the analysis. Based on standards from the National Institute on Alcohol Abuse and Alcoholism, moderate drinking was defined as between 1 and 7 drinks per week for females and between 1 and 14 drinks per week for males, whereas heavy drinking was defined as greater than 7 drinks for females and greater than 14 drinks for males per week (National Institute on Alcohol Abuse and Alcoholism, 2017). BMI, which was calculated from participant's weight in kilograms divided by the square of their height in meters, was categorized according to the World Health Organization guidelines as either normal (BMI <25 kg/m^2^), overweight (BMI: 25.0–29.9 kg/m^2^), obese class I (BMI: 30.0–34.9 kg/m^2^), or obese class II (BMI ≥35 kg/m^2^) (Weir and Jan 2024). If individuals did not have their measurement recorded during their interview, we used their self-reported weight and height.

### 2.7 Statistical analysis

#### 2.7.1 Correlation among chronological age, epigenetic clocks, and EAA

We first assessed the relationship between five epigenetic clocks (HorvathAge, HannumAge, PhenoAge, GrimAge, DunedinPACE) and chronological age using Pearson correlations. Additionally, we calculated the correlations among HorvathAge acceleration (HorvathAA), HannumAge acceleration (HannumAA), PhenoAge acceleration (PhenoAA), GrimAge acceleration (GrimAA), and DunedinPACE.

#### 2.7.2 Association between psychosocial factors/depressive symptoms and epigenetic age acceleration

Next, we examined the relationships between the exposures of interest (psychosocial factors, depressive symptoms) and each EAA measure separately using linear regression models adjusted for sex, educational attainment, marital status, having children, employment status, total wealth, year of psychosocial stress measurement, white blood cell proportions, and genetic ancestry PCs (Model 1). Further, because health behaviors may be on the causal pathway between psychosocial factors and epigenetic aging, we were interested in understanding the direct relationship between the exposures of interest and epigenetic aging independent of these health behaviors. Thus, as a secondary analysis, we adjusted the primary models for smoking status, alcohol use, and BMI (Model 2). To account for multiple testing, we applied a false discovery rate (FDR) correction using the Benjamini-Hochberg procedure, and associations with an FDR q < 0.05 were considered significant. Because we standardized all our exposures of interest, the effect sizes can be interpreted as the increase in EAA associated with a 1 SD increase in cumulative stress/loneliness/depressive symptoms.

#### 2.7.3 Effect modification by sex and educational attainment

We then assessed sex and educational attainment as possible effect modifiers of the relationship between the exposures of interest and epigenetic aging by including a multiplicative interaction term for each demographic factor in the linear regression models. To better characterize the effect of education on these relationships, we dichotomized educational attainment into two separate variables: 1) no degree vs. high school degree or higher and 2) less than college degree vs. college degree or higher (Lin et al., 2024). We only tested for interactions when both the exposure of interest and the demographic factor were associated with EAA in Model 1. For this analysis, p < 0.05 was considered significant.

#### 2.7.4 Mediation by depressive symptoms on associations between psychosocial factors and EAA

Finally, for the significant associations between psychosocial factors and EAA measures identified in Model 1, we conducted a formal mediation analysis using the *regmedint* package in R to examine the mediating effect of depressive symptoms on the association between psychosocial factors and EAA (Li et al., 2023b). This method is an extension of the regression-based causal mediation analysis using the counterfactual framework, which decomposes the total effect into the sum of the direct effect and indirect effect (Qin, 2024). In causal mediation analysis, the total, direct, and indirect effects are defined relative to a contrast in an exposure X, denoted as *x* vs. *x*∗, where *x*∗ is the reference level. Because both cumulative psychosocial stress and loneliness are standardized, we set *x*∗ = 0 and *x* = 1 to represent a change in one standard deviation of the psychosocial exposure. The total effect (TE) represents the change in an outcome, if the exposure were to change from *x*∗ to *x* for the entire population. The natural direct effect (NDE) expresses the change in an outcome from *x*∗ to *x*, allowing the mediator for each individual to be kept at the level it would have taken under exposure *x∗*. Finally, the natural indirect effect (NIDE) is the change in outcome, exclusively quantified by the exposure induced change of the mediator, meaning the exposure is controlled at *x*, but the mediator is changed from the level it would take at *x*∗ to the level it would take at *x* (VanderWeele and Vansteelandt, 2009).

In single mediator models with a continuous mediator and outcome, traditional mediation methods, including the difference and product method, provide similar effect estimates as causal mediation analysis. However, one advantage of causal mediation analysis is that it allows for exposure-mediator interactions, which capture any changes in the strength or direction of mediation by level of the exposure (Rijnhart et al., 2021). In this analysis, interactions between psychosocial factors and depressive symptoms were included in all mediation models.

We used linear regression models to estimate: (1) the association of each psychosocial factor with depressive symptoms (Equation (1)) and (2) the association of depressive symptoms with EAA, adjusting for the psychosocial factor and the psychosocial factor-depressive symptoms interaction (Equation (2)).

(Equation1) $$M=\alpha_{0}+\alpha_{1}X+\alpha_{C}^{T}C+\varepsilon_{M\;},$$

(Equation2) $$Y=\beta_{0}+\beta_{1}X+\beta_{2}M+\beta_{3}X*M+\beta_{C}^{T}C+\varepsilon_{Y}$$

In Equations (Equation1), (Equation2)), which reflect the mediator and outcome models for a single individual, M represents depressive symptoms, X represents the psychosocial factor (psychosocial stress, loneliness), C is the set of potential confounders (sex, educational attainment, marital status, having children, employment status, total wealth, year of psychosocial stress measurement, white blood cell proportions, and genetic ancestry PCs), Y represents a single EAA measure, X∗M represents the interaction between the psychosocial factor and depressive symptoms, and $\varepsilon_{M}$ and $\varepsilon_{Y}$ are residual errors, following an independent and normal distribution. Subsequently, TE, NDE, and NIDE, as well as the proportion mediated, were calculated for each exposure-outcome pair.

#### 2.7.5 Mediation stratified by sex and educational attainment

We then conducted stratified mediation analyses for any significant interactions observed between sociodemographic characteristics and psychosocial factors on EAA in the full sample. This allowed us to understand whether the mediation effect of depressive symptoms on the relationship between each psychosocial factor and EAA differed by sociodemographic characteristics. To ensure that our study was sufficiently powered to detected significant effects within the stratified sociodemographic groups, we performed a post-hoc power analysis using the Monte Carlo Power Analysis for Indirect Effects (Schoemann et al., 2017).

#### 2.7.6 Mediation sensitivity analysis after removing loneliness item from CES-D

Finally, one item in the CES-D explicitly asks participants the question, “Do you feel lonely?” (yes/no). Thus, as a sensitivity analysis, we removed that item when assessing the mediation effect of depressive symptoms on the relationship between loneliness and EAA to ensure that it was not significantly driving the mediation results. For all mediation analyses, p < 0.05 was considered significant.

## 3 Results

### 3.1 Descriptive statistics

Sample characteristics are shown in Table 1. The mean age of participants (n = 2681) was 70.4 years (SD: 9.5) and the majority were female (59 %). Approximately three-quarters of the sample were non-Hispanic White (74 %), 13 % were non-Hispanic Black, 10 % were Hispanic, and 3 % were another race/ethnicity. Over a quarter of participants had a college degree or higher (26 %) and nearly half were working for pay (44 %). Seventy percent of the sample was either married or had a partner, and the vast majority of participants had at least one child (89 %). In terms of health behaviors, 45 % of participants never smoked, 43 % were former smokers, and 12 % reported currently smoking at the time of their interview. A total of 59 % reported not drinking alcohol, while 34 % were moderate drinkers, and 7 % were heavy drinkers. Finally, approximately 80 % of the sample was classified as either overweight or obese, having a BMI greater than 25 kg/m^2^. Participants with complete data on stress and adjustment covariates tended to be older, have higher education, and have healthier behaviors than those from the DNA methylation sample who were excluded, though the effect sizes between the two groups were relatively small (Cramer's V and Cohen's d < 0.30 for all covariates) (Supplemental Table 4).

**Table 1:** Characteristics of Health and Retirement Study analytic sample (N = 2681).

Characteristic	N (%) or mean (SD)
Age, years	70.4 (9.5)
Female	1585 (59.2)
**Race/Ethnicity**	
Hispanic	270 (10.1)
Black	349 (13.0)
White	1992 (74.3)
Other	69 (2.6)
**Education**	
No degree	346 (12.9)
High school degree	1625 (60.6)
College degree or higher	710 (26.5)
**Employment Status**	
Working for pay	1173 (43.8)
Not working for pay	1508 (56.2)
**Marital Status**	
Married/partnered	1876 (70.0)
Separated/divorced/widowed	680 (25.4)
Never married	125 (4.7)
Have children	2384 (88.9)
Total Wealth	$443,612 ($954,116)
**Smoking Status**	
Never smoked	1205 (45.2)
Former smoker	1134 (42.6)
Current smoker	326 (12.2)
**Alcohol Use**	
Non-drinker	1582 (59.1)
Moderate drinker	912 (34.0)
Heavy drinker	186 (6.9)
**BMI (kg/m^2^)**	
Underweight**/**Normal	525 (19.6)
Overweight	896 (33.5)
Obese class I	728 (27.2)
Obese class II	524 (19.6)
**Epigenetic Age**	
HorvathAge, years	66.4 (9.5)
HannumAge, years	55.3 (9.2)
PhenoAge, years	58.1 (10.0)
GrimAge, years	68.6 (8.6)
**Epigenetic Age Acceleration (AA)**	
HorvathAA, years	0.0 (6.5)
HannumAA, years	0.0 (5.4)
PhenoAA, years	0.0 (6.9)
GrimAA, years	0.0 (4.4)
DunedinPACE, accelerated aging per year	1.03 (0.15)

### 3.2 Correlation among chronological age, epigenetic clocks, and EAA

Participants’ epigenetic age ranged from 55.3 to 68.6 years based on the four epigenetic clocks. Additionally, DunedinPACE had a mean of 1.03 (SD: 0.15), suggesting this sample had a slightly accelerated pace of aging. Correlations among the five epigenetic clocks were between 0.06 and 0.77, and each clock was highly correlated with chronological age (all p < 0.001; Supplemental Table 5). When adjusting the clocks for chronological age, the strength of the associations diminished, with correlations among EAA measures ranging from 0 to 0.62 (Supplemental Table 6). These correlations remained nominally significant (p < 0.05), aside from DunedinPACE and Horvath age acceleration.

### 3.3 Association between psychosocial factors/depressive symptoms and epigenetic age acceleration

In the primary analysis (Model 1), a 1-standard deviation (SD) increase in cumulative psychosocial stress was associated with accelerated aging in HannumAA (β = 0.33 years [0.14, 0.52]), PhenoAA (β = 0.42 years [0.16, 0.69]), and GrimAA (β = 0.42 years [0.26, 0.58]), as well as a faster pace of aging in DunedinPACE (β = 0.013 [0.007, 0.019]; Table 2). Similarly, a 1-SD increase in loneliness was associated with accelerated aging in HannumAA (β = 0.32 years [0.13, 0.51]), PhenoAA (β = 0.49 years [0.23, 0.75]), and GrimAA (β = 0.22 years [0.06, 0.37]), and a faster pace of aging in DunedinPACE (β = 0.01 [0.004, 0.015]). Finally, an increase in depressive symptoms was associated with higher HannumAA (β = 0.27 years [0.08, 0.46]), and GrimAA (β = 0.36 years [0.20, 0.53]), and DunedinPACE (β = 0.017 [0.012, 0.023]).

**Table 2:** Associations between psychosocial factors/depressive symptoms and epigenetic age acceleration.

	HorvathAA	HannumAA	PhenoAA	GrimAA^a^	DunedinPACE^b^
	β (95 % CI)	β (95 % CI)	β (95 % CI)	β (95 % CI)	β (95 % CI)
**Model 1 (N=2681)**					
Psychosocial Stress	−0.10 (−0.35, 0.15)	**0.33 (0.14, 0.53)**	**0.42 (0.15, 0.69)**	**0.41 (0.25, 0.58)**	**0.013 (0.007, 0.019)**
Loneliness	0.18 (−0.06, 0.42)	**0.32 (0.13, 0.51)**	**0.49 (0.23, 0.75)**	**0.21 (0.05, 0.37)**	**0.010 (0.004, 0.015)**
Depressive Symptoms	−0.004 (−0.25, 0.24)	**0.27 (0.08, 0.46)**	0.24 (−0.02, 0.50)	**0.36 (0.20, 0.52)**	**0.017 (0.012, 0.023)**
**Model 2 (N=2656)**					
Psychosocial Stress	−0.14 (−0.39,0.12)	**0.26 (0.07, 0.46)**	0.26 (−0.001, 0.53)	0.08 (−0.06, 0.22)	**0.007 (0.002, 0.012)**
Loneliness	0.14 (−0.10, 0.38)	**0.26 (0.07, 0.45)**	**0.41 (0.15, 0.67)**	0.08 (−0.06, 0.21)	**0.007 (0.002, 0.012)**
Depressive Symptoms	−0.05 (−0.30, 0.19)	0.20 (0.08, 0.40)	0.12 (−0.14, 0.38)	**0.16 (0.03, 0.30)**	**0.013 (0.008, 0.018)**

Model 1: Epigenetic age acceleration ∼ Psychosocial factor + Sex + Educational attainment + Marital status + Employment + Has child + Top 10 genetic ancestry PCs + WBC proportions + Year of psychosocial battery.

Model 2: Model 1 + Smoking status + Alcohol use + BMI.

β represents change in years of epigenetic age (or per-year acceleration of aging for DunedinPACE) for 1 SD increase in the mean psychosocial factor score. FDR q-value <0.05 is bolded.

a Models were not adjusted for sex, as GrimAge was regressed upon sex to calculate GrimAA.

b Age was included as an additional covariate in these models.

After including smoking status, alcohol use, and BMI as additional covariates (Model 2), we observed an attenuation of beta estimates for all three exposures (Table 2). However, the observed associations remained significant at an FDR q < 0.05, except for associations between cumulative stress and both PhenoAA and GrimAA, loneliness and GrimAA, and depressive symptoms and HannumAA.

### 3.4 Effect modification by sex and educational attainment

**Fig. 1:**
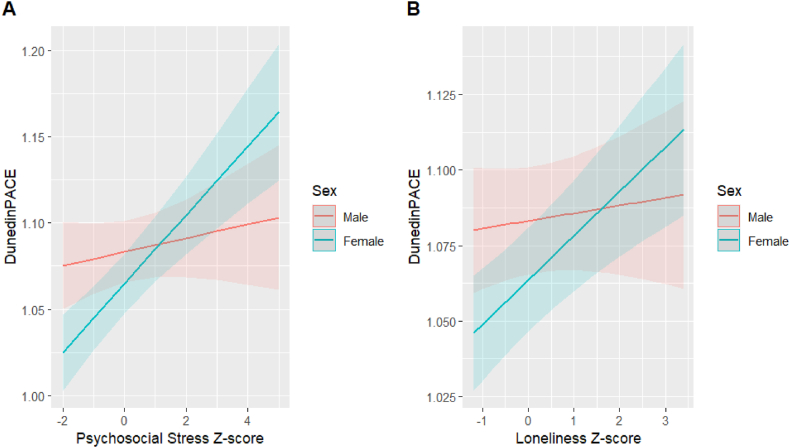
Plots of significant interactions between psychosocial factors and sex on DunedinPACE (p < 0.05). Predicted DunedinPACE values for (A) cumulative psychosocial stress z-scores and (B) loneliness z-scores by sex are shown with 95 % confidence intervals, which intend to capture the true line in the population 95 % of the time across repeated samples. Interaction models: DunedinPACE ∼ psychosocial factor + sex + educational attainment + marital status + employment + has child + top 10 genetic ancestry PCs + WBC proportion + year of psychosocial battery + psychosocial factor∗sex.

We next assessed whether the associations between our exposures of interest and EAA were modified by specific sociodemographic characteristics, including sex and educational attainment. We found significant interactions between sex and both cumulative psychosocial stress and loneliness on DunedinPACE (Table 3 and Fig. 1). A 1-SD increase in psychosocial stress was associated with a 0.019 increase in pace of aging for males versus a 0.036 increase in pace of aging for females. Similarly, a 1-SD increase in loneliness was associated with a 0.015 increase in pace of aging for males versus a 0.027 increase in pace of aging for females. These interactions remained significant after adjusting for Model 2 covariates (Supplemental Table 7).

**Table 3:** Two-way interaction between psychosocial factors/depressive symptoms and demographic characteristics on epigenetic age acceleration (Model 1; N = 2681).

Interaction Term	Exposure	EAA measure	β_Psychosocial_	P_Psychosocial_	β_Demographic_	P_Demographic_	β_Interaction_	P_Interaction_
*Exposure∗Sex*	Psychosocial Stress	Hannum	0.15	0.31	**−1.07**	**1.07E-07**	0.33	0.08
		DunedinPACE	0.004	0.33	**−0.02**	**8.34E-04**	**0.016**	**0.004**
	Loneliness	Hannum	**0.31**	**0.03**	**−1.08**	**9.34E-08**	0.01	0.94
		DunedinPACE	0.003	0.53	**−0.02**	**4.02E-04**	**0.01**	**0.003**
	Depressive Symptoms	Hannum	0.20	0.23	**−1.18**	**5.91E-09**	0.11	0.59
		DunedinPACE	**0.012**	**0.01**	**−0.02**	**2.47E-05**	0.008	0.13
*Exposure∗College Degree*	Psychosocial Stress	PhenoAge	**0.49**	**0.001**	**−0.64**	**0.04**	−0.32	0.29
		GrimAge	**0.44**	**2.84E-06**	**−1.54**	**1.07E-15**	−0.08	0.66
		DunedinPACE	**0.01**	**1.46E-05**	**−0.04**	**1.99E-10**	−0.002	0.73
	Loneliness	PhenoAge	**0.66**	**1.59E-05**	**−0.68**	**0.03**	**−0.60**	**0.04**
		GrimAge	**0.32**	**5.68E-04**	**−1.62**	**2.00E-16**	**−0.35**	**0.049**
		DunedinPACE	**0.01**	**1.28E-05**	**−0.04**	**9.42E-12**	**−0.01**	**0.03**
	Depressive Symptoms	GrimAge	**0.42**	**2.70E-06**	**−1.56**	**1.28E-15**	−0.21	0.31
		DunedinPACE	**0.02**	**1.57E-10**	**−0.04**	**6.46E-10**	−0.004	0.56

Interaction Model: Epigenetic age acceleration ∼ Psychosocial factor + Sex + Educational attainment + Marital status + Employment + Has child + Top 10 genetic ancestry PCs + WBC proportion + Year of psychosocial battery + Psychosocial factor∗Demographic characteristic.

βPsychosocial represents change in years of epigenetic age (or per-year acceleration of aging for DunedinPACE) for 1 SD increase in the mean psychosocial factor score; βDemographic represents change in years of epigenetic age (or per-year acceleration of aging for DunedinPACE) for females/those with a college degree; βInteraction represents difference in years of epigenetic age (or per-year acceleration of aging for DunedinPACE) for 1 SD increase in mean psychosocial factor score between levels of demographic variables.

We only tested for interactions when the main effects of both the exposure of interest (psychosocial stress, loneliness, depressive symptoms) and demographic factor were associated with EAA in Model 1.

P-value <0.05 is bolded.

**Fig. 2:**
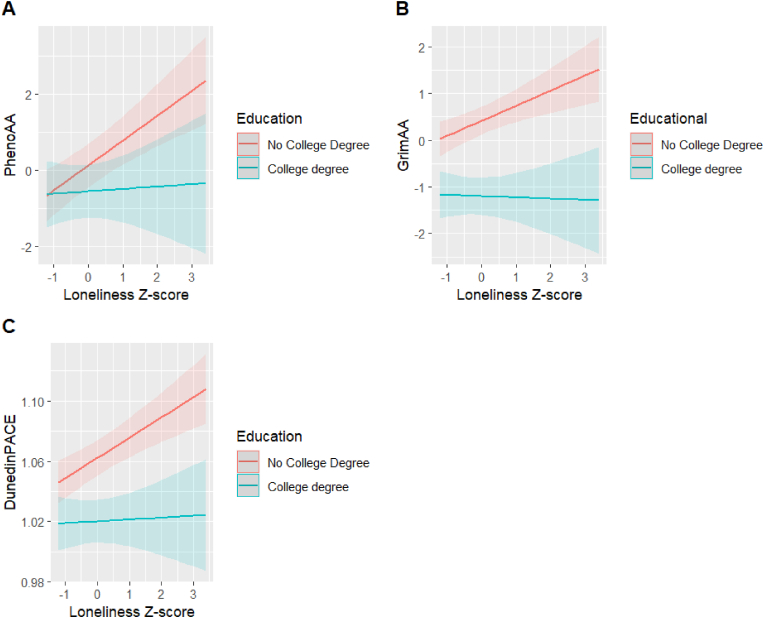
Plots of significant interactions between loneliness and educational attainment on epigenetic age acceleration measures (p < 0.05). Predicted (A) PhenoAA, (B) GrimAA, and (C) DunedinPACE values for loneliness z-scores by educational attainment (no college degree vs. college degree or higher) are shown with 95 % confidence intervals. Interaction models: EAA measure ∼ loneliness z_score + sex + educational attainment + marital status + employment + has child + top 10 genetic ancestry PCs + WBC proportion + year of psychosocial battery + loneliness z_score∗educational attainment.

Additionally, after dichotomizing educational attainment to be less than college degree vs. college degree or higher, we observed significant interactions between loneliness and educational attainment on EAA in PhenoAge and GrimAge, and on DunedinPACE (Table 3 and Fig. 2). A 1-SD increase in loneliness was associated with a 0.061 increase and 0.027 decrease in PhenoAA and GrimAA, respectively, for those with a college degree, as opposed to a 0.66 increase in PhenoAA and a 0.32 increase in GrimAA for those without a college degree. Additionally, a 1-SD increase in loneliness was associated with a 0.001 versus a 0.013 increase in pace of aging for individuals with and without a college degree, respectively, as measured by DunedinPACE. In Model 2, these interactions were no longer significant (Supplemental Table 7). When dichotomizing educational attainment to be no degree vs. high school degree or higher, we did not observe significant interactions for any of the EAA measures (Supplementary Table 8).

### 3.5 Mediation by depressive symptoms on associations between psychosocial factors and EAA

Finally, we investigated the mediating effect of depressive symptoms on the associations between psychosocial factors and EAA. Cumulative psychosocial stress explained between 1.5 % and 4.7 % of the total variability of HannumAA, PhenoAA, GrimAA, and DunedinPACE after adjusting for sociodemographic covariates in the primary model (Model 1), and between 0.1 % and 0.9 % of the total variability after further adjusting for health behaviors (Model 2; Supplemental Table 9). Loneliness explained between 1.4 % and 4.1 % of the total variability in Model 1 and between 0.1 % and 2.4 % of the total variability in Model 2. We found that depressive symptoms mediated 25.1 %, 24.0 %, and 35.3 % of the relationship between cumulative psychosocial stress and HannumAA, GrimAA, and DunedinPACE, respectively (Table 4). Furthermore, depressive symptoms mediated 39.9 % of the relationship between loneliness and GrimAA, as well as 36.5 % of the relationship between loneliness and DunedinPACE (all p < 0.05). After further adjusting for health behaviors in the mediation models, depressive symptoms mediated 49.9 % of the association between cumulative stress and DunedinPACE, and 33.2 % of the association between loneliness and DunedinPACE (Supplemental Table 10).

**Table 4:** Mediation by depressive symptoms of the relationships between psychosocial stress/loneliness and epigenetic age acceleration (Model 1; N = 2681).

	HannumAA		PhenoAA		GrimAA		DunedinPACE	
	β (95 % CI)	P-value	β (95 % CI)	P-value	β (95 % CI)	P-value	β (95 % CI)	P-value
**Psychosocial Stress**								
Total Effect	**0.31 (0.12, 0.51)**	**0.002**	0.44 (0.17, 0.71)	0.001	**0.40 (0.24, 0.57)**	**1.65E-06**	**0.013 (0.007, 0.019)**	**7.04E-06**
Direct Effect	**0.23 (0.02, 0.44)**	**0.028**	0.42 (0.14, 0.71)	0.004	**0.31 (0.13, 0.48)**	**5.53E-04**	**0.008 (0.002, 0.014)**	**0.006**
Indirect Effect rowhead	0.08 (0.01, 0.14)	0.016	0.02 (−0.07, 0.10)	0.75	0.09 (0.04, 0.15)	4.09E-04	0.004 (0.003, 0.006)	1.30E-06
Proportion Mediated	**0.25**	**--**	0.03	**--**	**0.24**	**--**	**0.35**	**--**
**Loneliness**								
Total Effect	0.32 (0.13, 0.51)	9.06E-04	0.50 (0.24, 0.76)	1.61E-04	**0.21 (0.05, 0.37)**	**0.008**	**0.010 (0.005, 0.015)**	**2.85E-04**
Direct Effect	0.26 (0.06, 0.46)	0.01	0.49 (0.22, 0.76)	4.39E-04	**0.13 (-0.04, 0.29)**	**0.13**	**0.006 (0.0007, 0.011)**	**0.03**
Indirect Effect	0.06 (−0.0007, 0.11)	0.05	0.01 (−0.07, 0.09)	0.81	**0.09 (0.04, 0.13)**	**5.49E-04**	**0.004 (0.002, 0.005)**	**1.26E-05**
Proportion Mediated	0.18	**--**	0.02	**--**	**0.40**	**--**	**0.36**	**--**

Associations with significant indirect effects (p-value <0.05) are bolded.

### 3.6 Mediation stratified by sex and educational attainment

We then conducted stratified mediation analyses when significant interactions between sociodemographic characteristics and psychosocial factors on EAA were present. Specifically, we examined the mediating effect of depressive symptoms on the relationship between psychosocial factors and DunedinPACE stratified by sex. In females, depressive symptoms mediated 27.6 % of the relationship between cumulative stress and DunedinPACE, as well as 24.8 % of the relationship between loneliness and DunedinPACE (Model 1; Supplemental Table 11). After adjusting for health behaviors, the mediation effects persisted, with depressive symptoms mediating 30.6 % and 19.9 % of the associations between cumulative stress and loneliness, respectively, and DunedinPACE. (Supplemental Table 11). Conversely, in males, the total effects of both cumulative stress and loneliness on DunedinPACE were not significant in Model 1. As a result, the mediation effect of depressive symptoms on these relationships was not evaluated.

Additionally, we examined the mediating effect of depressive symptoms on the associations between loneliness and PhenoAA, GrimAA, and DunedinPACE stratified by educational attainment (college degree vs. no college degree). For those without a college degree, depressive symptoms mediated 36.5 % and 31.1 % of the relationships between loneliness and both GrimAA and DunedinPACE, respectively (Model 1; Supplemental Table 12). However, the total effects of loneliness on GrimAA and DunedinPACE were no longer significant after adjusting for health behaviors (Model 2). For those with a college degree, the total effects of loneliness on all EAA measures were not significant in Model 1. Post-hoc power analysis indicated that we had 90–99 % power to detect the effects within sociodemographic groups, suggesting that our analyses were sufficiently powered even after stratifying our sample (Supplemental Tables 11–12).

### 3.7 Mediation sensitivity analysis after removing loneliness item from CES-D

Finally, we assessed the mediating effect of depressive symptoms on the association between loneliness and EAA, excluding the loneliness-specific item from the CES-D. Results obtained for both Models 1 and 2 were substantively similar to results from the primary mediation analysis (Supplemental Table 13).

## 4 Discussion

In this study, psychosocial factors, including cumulative psychosocial stress and loneliness, and depressive symptoms were associated with EAA in a multi-racial/ethnic cohort of older adults. We found that females, as well as those with no college degree, were more sensitive to the effects of psychosocial factors on epigenetic aging. Furthermore, depressive symptoms partially mediated the relationship between psychosocial factors and EAA for a number of epigenetic clocks. To our knowledge, this was the first study to examine the mediating role of mental health on the relationships between cumulative psychosocial stress, loneliness, and epigenetic aging in a diverse sample of older adults.

Several studies examined the association between individual psychosocial stress measures and epigenetic aging; however, results were mixed. In the Berlin Aging Study II (BASE-II), a cohort of middle-aged and older adults comprised mostly of European ancestry, Vetter et al. (2022) found that psychosocial stress, evaluated using the Perceived Stress Scale, was not associated with HorvathAA, HannumAA, PhenoAA, and GrimAA (Vetter et al., 2022). Skinner et al. (2023) looked at those same EAA measures in a cohort of postmenopausal women from the Women's Health Initiative (WHI) and only observed associations between stressful life events and GrimAA (Skinner et al., 2024). A systematic review by Lim et al. (2022) provided further evidence of discordant findings when they reported that only two of the six studies included in the review had positive associations between either lifetime trauma or discrimination and EAA (Lim et al., 2022). A possible explanation for why we are observing stronger associations between psychosocial stress and several EAA measures in our study is because the psychosocial stress measure captures multiple stress domains across the life course. Research has shown that the accumulation of stress results in worsened physical and mental health outcomes compared to individual stressors (Haight et al., 2023; Slopen et al., 2018). Thus, it is reasonable to assume cumulative psychosocial stress has a similar dose-response relationship on epigenetic aging. Future analyses should examine the associations between individual stress domains and EAA in the same sample to assess if particular domains are driving the relationships.

In terms of loneliness, findings from this study are fairly consistent with those from previous literature. In a cohort of middle-aged adults from the Midlife in the United States (MIDUS) study, Freilich et al. (2024) found that loneliness, measured by a single, self-reported item, was associated with EAA estimated by GrimAge and DunedinPACE after adjusting for sex, race and education (Freilich et al., 2024). The addition of behavioral characteristics, including smoking status, alcohol use, and BMI, attenuated their results, though the associations did remain nominally significant (p < 0.05). Although associations with HannumAA and PhenoAA were not significant, as they were in our study, the beta coefficients were in the same direction. Similarly, Beach et al. (2022) examined the association between loneliness and DunedinPACE in a sample of middle-aged Black adults using two items from the UCLA Loneliness Scale (Beach et al., 2022). Over an 11-year period, they found that change in loneliness was associated with a change in DunedinPACE, after adjusting for sociodemographic characteristics, as well as smoking and alcohol use.

When we assessed sociodemographic characteristics as possible effect modifiers of the relationship between the psychosocial factors and epigenetic aging, interactions revealed that the effects of psychosocial stress and loneliness on epigenetic aging measured by DunedinPACE were driven by females. Prior research has determined differences in stress response between males and females due to a variety of biological and psychological mechanisms (Verma et al., 2011; Goldfarb et al., 2019; Brivio et al., 2020). Two functional magnetic resonance imaging (MRI) studies performed by Goldstein et al. (2010) and Cohen et al. (2023) demonstrated that response to stress activated different regions of the brain for men and women (Goldstein et al., 2010; Cohen et al., 2023). Specifically, they found that sex differences in stress were regulated through hormones via the impact of subcortical brain activity. Further, female sex hormones were found to attenuate sympathoadrenal and hypothalamic-pituitary-adrenal (HPA) responsiveness within the endocrine and nervous systems, leading to delayed cortisol feedback on the brain and lessened containment of the stress response (Verma et al., 2011). As a result, the weakened cortisol feedback effects on the HPA could prompt prolonged activation of the stress-response system in females, resulting in negative health consequences, including coronary heart disease (CHD), type 2 diabetes, and metabolic syndrome (Kajantie and Phillips, 2006). Finally, from a psychological perspective, females are often less resilient to stress (Yalcin-Siedentopf et al., 2021; Lowe et al., 2021) and have stronger perceptions of stress (Costa et al., 2021; Xu et al., 2015) than their male counterparts. This may partially explain why females experience higher rates of mental health disorders, including anxiety (Bahrami and Yousefi, 2011; Farhane-Medina et al., 2022) and depression (Maciejewski et al., 2001; Abate, 2013). Thus, results from our study, which found that females experienced a sharper acceleration in epigenetic aging in the presence of stress, are logical in the context of this research.

Similarly, differences in the effects of loneliness on PhenoAge, GrimAge, and DunedinPACE by educational attainment were observed, where individuals without a college degree were driving the associations. This is consistent with other studies, which have shown that psychosocial distress is associated with poorer health outcomes and mortality in lower SES groups (Lazzarino et al., 2013; Talala et al., 2011; McLachlan and Gale, 2018). One possible explanation for these findings is that higher-educated individuals have a larger social support network and have a greater abundance of resources available to help cope with stressful events (Matthews and Gallo, 2011). Results from our study and others warrant the need for future research to better understand why health disparities exist in the context of psychosocial stress.

Mediation analyses revealed that depressive symptoms mediated between 24 % and 35 % of the relationship between cumulative psychosocial stress and HannumAA, GrimAA, and DunedinPACE, in addition to 40 % and 37 % of the relationships between loneliness and GrimAA and DunedinPACE, respectively. When significant interactions between sociodemographic characteristics and psychosocial factors on EAA were present, we performed stratified mediation analyses and found that the mediation effects persisted only in females and those without a college degree. A study by Beydoun et al. (2022), which examined the mediating role of depressive symptoms on the associations between perceived discrimination and epigenetic aging in HRS, yielded similar results (Beydoun et al., 2022). After adjusting the models for birth cohort, sex, and race/ethnicity, they found that depressive symptoms mediated roughly 39 % and 32 % of the relationships between reasons for perceived discrimination (RPD) and GrimAge and DunedinPoAm38, a pace of aging measure that preceded DunedinPACE, respectively. Further, when stratified by sex, the mediating effects of depressive symptoms on the associations between RPD and these two epigenetic clocks were only present in females. As both of these studies were conducted in HRS, a future next step would be to replicate these findings in an independent cohort of older adults.

One potential mechanism through which depressive symptoms may influence accelerated epigenetic aging involves altered neural and physiological responses to stress. These altered responses, which are often observed in depression, impact an array of systems including HPA axis function, the immune system, and brain function. Under normal circumstances, a stressful event triggers the release of cortisol in response to the stressor before returning the stress-response systems to usual levels, achieving allostasis (Szymkowicz et al., 2023). However, when faced with prolonged stress and depressive episodes, the stress-response systems continuously interact, resulting in elevated cortisol levels, increased allostatic load, and chronic, low-grade inflammation (Berk et al., 2013). Over time, the long-term activation of these systems contributes to accelerated epigenetic aging, disrupting the development of brain architecture and leading to regional brain atrophy and reduced neurotrophic function (Geerlings et al., 2015; Lupien et al., 1998; Cox et al., 2015). Several longitudinal studies have supported this hypothesis, finding that persistent depression was associated with increased white matter hyperintensity volume and greater hippocampal volume decline (Taylor et al., 2003, Taylor et al., 2014). Further, evidence has shown that clinical depression may result in increased oxidative and nitrosative stress (O&NS), defined as an imbalance between the production of reactive oxygen and nitrogen species and the body's ability to counteract them with antioxidants (Moylan et al., 2013; Leonard and Maes, 2012). Consequences of long-term O&NS include damage to proteins, lipids, and DNA, as well as epigenetic alterations, resulting in accelerated epigenetic aging and age-related diseases (Wu et al., 2024; Guillaumet-Adkins et al., 2017; Morris et al., 2016).

The strength of associations between psychosocial factors and EAA varied across epigenetic clocks. We observed the weakest associations with first-generation clocks, though psychosocial factors and depressive symptoms were associated with HannumAA. Alternatively, cumulative psychosocial stress, loneliness, and depressive symptoms were strongly associated with second- and third-generation clocks. A systematic review conducted by Oblak et al. (2021), which examined general trends regarding the associations between epigenetic clocks and several biological, social and environmental factors, supported our findings (Oblak et al., 2021). This review concluded that differences between chronological age-trained (first-generation) clocks and mortality-trained (second- and third-generation) clocks were more prominent in mental health outcomes, including post-traumatic stress disorder, schizophrenia, and depression, than physical outcomes. Thus, future research should focus on exploring distinct biological mechanisms that may link individual epigenetic clocks to mental health outcomes.

There are limitations to this study that must be acknowledged. First, psychosocial factors, depressive symptoms, and epigenetic aging were all assessed at a single time point. Although we implemented a longitudinal study design that established temporality between the exposures, mediator, and outcomes, these variables were only measured four to six years apart. In light of this, reverse causation is possible, where epigenetic aging may instead influence psychosocial factors and depressive symptoms. Furthermore, cumulative stress, loneliness, and depressive symptoms are likely to be correlated across time. Having only one time point available for each measure limits our ability to demonstrate that depressive symptoms mediated the relationship between psychosocial factors and EAA, as opposed to confounding those associations. Future studies would benefit from examining these variables at multiple time points to assess changes in epigenetic aging resulting from psychosocial factors, as well as the mediating effect of depressive symptoms. Incorporating a longitudinal study design with repeated measures would better establish temporality and allow for causal inferences.

Additionally, although the CES-D shows high diagnostic accuracy and is often used as a first step in screening for depression (Park and Yu, 2021), this subjective measure assesses depressive symptoms over a short period of time and does not represent a diagnosis of clinical depression. Therefore, these findings should be replicated in a population that has clinical depression diagnoses available. Furthermore, the CES-D, as well as the psychosocial stress and loneliness scales, are all self-reported measures. Thus, they may be subject to information biases, including social desirability and recall bias. In this study, we would expect this to lead to non-differential misclassification of the exposure, as we do not anticipate that an individual's epigenetic age would influence the misclassification of their psychosocial factors or depressive symptoms. If present, this non-differential misclassification would likely bias our results toward the null, underestimating the true associations and resulting in conservative estimates of the relationships between psychosocial factors, depressive symptoms, and EAA (Haine et al., 2018).

Finally, when we stratified the mediation models by sex and educational attainment, we observed different sample sizes in each of the strata, which may have impacted the magnitude of the total effects. As a result, these analyses should be replicated in a population with larger strata to ensure the lack of association in certain sociodemographic groups was not due to statistical power.

This study also had several strengths. First, we examined the association between psychosocial factors and epigenetic aging using validated, self-report measures of loneliness and depressive symptoms, as well as a cumulative psychosocial stress score that comprised several domains of stress across the life course. Examining multiple psychosocial measures allowed us to compare the strength in which specific factors influenced epigenetic aging. Second, we implemented a causal mediation method that accounted for interactions between psychosocial factors and depressive symptoms in the mediation models. Thus, unlike more traditional mediation methods, this method captured differences in the strength of the mediation effect by levels of psychosocial stress and loneliness. Finally, this research was conducted in a sample comprised of Africans, Europeans, and Hispanics, which allowed us to examine the associations between psychosocial factors and epigenetic aging, as well as the mediating role of depressive symptoms on those relationships, in a diverse population of older adults.

From a clinical perspective, results from this study emphasize the increased need for psychosocial evaluation early on, particularly for women and individuals of low socioeconomic status, who may otherwise lack access to adequate mental health resources. These evaluations could be implemented as more frequent or involved screenings during annual wellness exams with the goal of identifying negative psychosocial factors before they lead to more serious mental health consequences. As this is preliminary evidence from a single study, more work needs to be done to validate our findings in diverse populations across the U.S.

## 5 Conclusion

Results from this study demonstrate that cumulative psychosocial stress, loneliness, and depressive symptoms are associated with epigenetic aging in a multi-racial/ethnic population of older adults, with females and those without a college degree appearing more sensitive to these effects. Furthermore, depressive symptoms partially mediate the relationship between psychosocial factors and epigenetic aging. Future research should be conducted to replicate these findings and further characterize the biological mechanisms through which psychosocial factors influence mental health outcomes and may contribute to age-related disease.

## CRediT authorship contribution statement

**Lauren A. Opsasnick:** Writing – review & editing, Writing – original draft, Funding acquisition, Formal analysis, Data curation, Conceptualization. **Wei Zhao:** Writing – review & editing. **Lauren L. Schmitz:** Writing – review & editing. **Scott M. Ratliff:** Writing – review & editing, Formal analysis. **Jessica D. Faul:** Writing – review & editing, Supervision. **Xiang Zhou:** Writing – review & editing, Supervision, Formal analysis. **Belinda L. Needham:** Writing – review & editing, Supervision. **Jennifer A. Smith:** Writing – review & editing, Supervision, Funding acquisition, Formal analysis, Conceptualization.

## Funding

The 10.13039/100005859HRS is sponsored by the 10.13039/100000049National Institute on Aging (grant number 10.13039/100000049NIA U01AG009740) and is conducted by the 10.13039/100007270University of Michigan. This analysis was supported by the National Heart Lung and Blood Institute (10.13039/100000050NHLBI, R01 HL141292 (Smith)) and 10.13039/100000051National Human Genome Research Institute (NHRGI, T32 HG000040 (Opsasnick 10.13039/100017573Rogers)).

## Declaration of competing interest

The authors declare that they have no known competing financial interests or personal relationships that could have appeared to influence the work reported in this paper.

## Data Availability

Data will be made available on request.
