# Supplementary material for: Depressive symptoms partially mediate the relationship between psychosocial factors and epigenetic age acceleration in a multi-racial/ethnic sample of older adults
Source: Brain Behav Immun Health. 2025 Apr 12;45:100994. doi: 10.1016/j.bbih.2025.100994 (PMC12022486; doi:10.1016/j.bbih.2025.100994)
Supplement: Multimedia component 1 [file mmc1.docx]

| Supplemental Table 1: Psychosocial stress measures included in the 6 stress domains. | | |
| --- | --- | --- |
| **Stress Domain** | **Stress Measure** | **Individual Items** |
| **Acute Life Events** | Lifetime Traumas | (1) Has experienced the death of child; (2) has ever been in a major fire, flood, earthquake or other natural disaster; (3) has ever fired a weapon in combat or been fired upon in combat; (4) has a spouse, partner, or child has ever been addicted to drugs or alcohol; (5) was a victim of a serious physical attack or assault; (6) has ever had a life-threatening illness or accident; (7) has spouse or a child has ever had a life-threatening illness or accident. |
|  | Stressful Life Events in Past 5 Years | (1) Has involuntarily lost a job for reasons other than retirement; (2) has been unemployed and looking for work for longer than 3 months; (3) was anyone in household unemployed and looking for work for longer than 3 months; (4) have moved to a worse residence or neighborhood; (5) have been robbed or home has been burglarized; (6) has been victim of fraud. |
| **Financial Stress** | Financial Strain | (1) Difficulty meeting monthly payments on bills; (2) distress caused by ongoing financial strain. |
|  | Lack of Financial Autonomy | (1) Satisfaction with his/her current financial situation; (2) amount of control over his/her financial situation (*reverse-coded*). |
| **Neighborhood Stress** | Neighborhood Disorder | (1) Vandalism and graffiti are a big problem in this area; (2) People would be afraid to walk alone in this area after dark; (3) This area is always full of rubbish and litter; (4) There are many vacant or deserted houses or storefronts in this area. |
| **Relationship Stress** | Marital Stressors | (1) How often do they make too many demands on you? (2) How much do they criticize you? (3) How much do they let you down when you are counting on them? (4) How much do they get on your nerves? |
|  | Child-Related Stressors |  |
|  | Other Family-Related |  |
|  | Friend Stressors |  |
| **Lifetime Discrimination** | Major Discriminatory Events | (1) Have been unfairly dismissed from a job; (2) have been unfairly not hired for a job; (3) have been unfairly denied a promotion; (4) have been unfairly prevented from moving into a neighborhood because the landlord or a realtor refused to sell or rent you a house or apartment; (5) have been unfairly denied a bank loan; (6) have been unfairly stopped, searched, questioned, physically threatened or abused by the police. |
|  | Perceived Everyday Discrimination | (1) Have been treated with less courtesy or respect than other people; (2) have received poorer service than other people at restaurants or stores; (3) people act as if they think you are not smart; (4) people act as if they are afraid of you; (5) you are threatened or harassed. |
| **Childhood Adversity** | Lifetime Traumas Before Age 18 | (1) Had to do a year of school over again; (2) was ever in trouble with the police; (3) either of his/her parents drank or used drugs so often that it caused problems in the family; (4) was ever physically abused by his/her parents. |
| *Note*. From “Epigenome-wide association study of long-term psychosocial stress in older adults,” by LO. Opsasnick, 2024, *Epigenetics*, *19*(1), p. 2 (Supplemental Material) (<https://doi.org/10.1080/15592294.2024.2323907>). CC BY 4.0. | | |

Supplemental Table 2. Eleven-item scale of loneliness derived from the 20-item Revised UCLA Loneliness Scale.

| The next questions are about how you feel about different aspects of your life. HOW MUCH OF THE TIME DO YOU FEEL... |
| --- |
| 1. You lack companionship? |
| 1. Left out? |
| 1. Isolated from others? |
| 1. That you are “in tune” with the people around you? |
| 1. Alone? |
| 1. That there are people you can talk to? |
| 1. That there are people you can turn to? |
| 1. That there are people who really understand you? |
| 1. That there are people you feel close to? |
| 1. Part of a group of friends? |
| 1. That you have a lot in common with the people around you? |
| **Coding:** 1 = Often, 2 = Some of the time, 3 = Hardly ever or never  **Scaling:** The index of loneliness was created by reverse coding items 1, 2, 3, and 5 and averaging the scores across all 11 items. The final loneliness score was set to missing if there were greater than 5 items with missing values. |
|  |

Supplemental Table 3. Eight-item scale of depressive symptoms derived from the Center for Epidemiologic Studies Depression Scale (CES-D).

| Now think about the past week and the feelings you have experienced. Please tell me if each of the following was true for you much of the time during the past week. Much of the time during the past week….. |
| --- |
| 1. You felt depressed? |
| 1. You felt that everything you did was an effort? |
| 1. Your sleep was restless? |
| 1. You were happy. |
| 1. You felt lonely. |
| 1. You enjoyed life. |
| 1. You felt sad? |
| 1. You could not get going? |
| **Coding:** 0=No, 1=Yes  **Scaling:** The depressive symptoms score was created by reverse coding items 4 and 6 and summing the scores across all 8 items. |

| Supplemental Table 4. Participant characteristics comparing those included and excluded in the primary analysis from the full DNA methylation sample (N=4018) |
| --- |

| **Characteristics** | **Included (n=2681)**  **N(%) or mean (SD)** | **Excluded (n=1337)**  **N(%) or mean (SD)** | **Effect Size**  **(Cohen’s d or Cramer’s V)** | **P-value** |
| --- | --- | --- | --- | --- |
| Age, years | 70.4 (9.5) | 67.5 (9.6) | 0.30 | <0.001 |
| Female | 1585 (59.2) | 764 (57.1) | 0.02 | 0.20 |
| **Race/Ethnicity** |  |  |  |  |
| Hispanic | 270 (10.1) | 297 (22.2) | 0.24 | <0.001 |
| Black | 349 (13.0) | 309 (23.1) |  |  |
| White | 1992 (74.3) | 677 (50.7) |  |  |
| Other | 69 (2.6) | 53 (4.0) |  |  |
| **Education** |  |  |  |  |
| No degree | 346 (12.9) | 329 (24.6) | 0.15 | <0.001 |
| HS degree | 1625 (60.6) | 750 (56.1) |  |  |
| College degree or higher | 710 (26.5) | 257 (19.2) |  |  |
| **Employment Status** |  |  |  |  |
| Working for pay | 1173 (43.8) | 919 (71.2) | 0.26 | <0.001 |
| Not working for pay | 1508 (56.2) | 371 (28.8) |  |  |
| Married/Partnered | 1876 (70.0) | 818 (63.3) | 0.07 | <0.001 |
| Have Children | 2384 (88.9) | 1176 (88.0) | 0.07 | 0.40 |
| Total Household Wealth ($) | $443,612 ($954,116) | $303,758 ($793.648) | 0.15 | <0.001 |
| **Smoking Status** |  |  |  |  |
| Never smoker | 1205 (45.2) | 542 (42.1) | 0.10 |  |
| Former smoker | 1134 (42.6) | 527 (40.9) |  | <0.001 |
| Current smoker | 326 (12.2) | 219 (17.0) |  |  |
| **Alcohol Use** |  |  |  |  |
| Never drinker | 1582 (59.1) | 832 (64.5) |  |  |
| Moderate drinker | 912 (34.0) | 367 (28.5) | 0.06 | 0.002 |
| Heavy drinker | 186 (6.9) | 91 (7.1) |  |  |
| BMI (kg/m^2^) | 30.4 (6.7) | 29.2 (6.5) | 0.18 | <0.001 |

Supplemental Table 5. Pearson correlation among epigenetic clocks and chronological age.

| **Epigenetic Age** | Age | HorvathAge | HannumAge | PhenoAge | GrimAge | DunedinPACE |
| --- | --- | --- | --- | --- | --- | --- |
| Age | 1.00 |  |  |  |  |  |
| HorvathAge | 0.72 | 1.00 |  |  |  |  |
| HannumAge | 0.81 | 0.77 | 1.00 |  |  |  |
| PhenoAge | 0.72 | 0.64 | 0.76 | 1.00 |  |  |
| GrimAge | 0.84 | 0.63 | 0.75 | 0.74 | 1.00 |  |
| DunedinPACE | 0.12 | 0.06 | 0.22 | 0.35 | 0.44 | 1.00 |

All correlations are significant at p<0.001.

Supplemental Table 6. Pearson correlation among epigenetic age acceleration measures.

| **Epigenetic Age Acceleration** | HorvathAA | HannumAA | PhenoAA | GrimAA | DunedinPACE |
| --- | --- | --- | --- | --- | --- |
| HorvathAA | 1.00 |  |  |  |  |
| HannumAA | 0.46 | 1.00 |  |  |  |
| PhenoAA | 0.25 | 0.43 | 1.00 |  |  |
| GrimAA | 0.04 | 0.17 | 0.35 | 1.00 |  |
| DunedinPACE | -0.03 | 0.21 | 0.38 | 0.62 | 1.00 |

All correlations are significant at p<0.05 except for DunedinPACE and HorvathAA (p=0.091).

| Supplemental Table 7. Two-way interaction between psychosocial factors/depressive symptoms and demographic characteristics on epigenetic age acceleration (Model 2; N=2,656). | | | | | | | | |
| --- | --- | --- | --- | --- | --- | --- | --- | --- |
| **Interaction Term** | **Exposure** | **EAA measure** | **β_Psychosocial_** | **P_Psychosocial_** | **β_Demographic_** | **P_Demographic_** | **β_Interaction_** | **P_Interaction_** |
| *Exposure*Sex* | Psychosocial Stress | Hannum | 0.06 | 0.66 | **-1.08** | **1.75E-07** | 0.35 | 0.06 |
|  |  | DunedinPACE | -0.001 | 0.71 | -0.009 | 0.09 | **0.02** | **0.002** |
|  | Loneliness | Hannum | 0.23 | 0.11 | **-1.07** | **2.34E-07** | 0.05 | 0.77 |
|  |  | DunedinPACE | 0.004 | 0.91 | -0.009 | 0.10 | **0.01** | **0.019** |
|  | Depressive Symptoms | Hannum | 0.13 | 0.42 | **-1.15** | **2.73E-08** | 0.10 | 0.60 |
|  |  | DunedinPACE | 0.007 | 0.09 | **-0.012** | **0.03** | 0.009 | 0.09 |
| *Exposure*College Degree* | Psychosocial Stress | PhenoAge | **0.33** | **0.03** | -0.49 | 0.11 | -0.30 | 0.33 |
|  |  | GrimAge | 0.06 | 0.44 | **-0.93** | **1.30E-08** | 0.10 | .0.55 |
|  |  | DunedinPACE | **0.007** | **0.02** | **-0.03** | **2.39E-05** | -0.001 | 0.84 |
|  | Loneliness | PhenoAge | **0.56** | **2.51E-04** | -0.51 | 0.10 | -0.52 | 0.07 |
|  |  | GrimAge | 0.14 | 0.07 | **-0.97** | **2.14E-09** | -0.23 | 0.13 |
|  |  | DunedinPACE | **0.01** | **7.48E-04** | **-0.03** | **7.86E-05** | -0.009 | 0.11 |
|  | Depressive Symptoms | GrimAge | **0.18** | **0.02** | **-0.93** | **2.02E-08** | -0.05 | 0.78 |
|  |  | DunedinPACE | **0.01** | **1.18E-06** | **-0.02** | **8.34E-05** | 0.0001 | 0.99 |
| Interaction Model: Epigenetic age acceleration ~ Psychosocial factor + Sex + Educational attainment + Marital status + Employment + Has child + Smoking status + Alcohol use + BMI + Top 10 genetic ancestry PCs + WBC proportion + Year of psychosocial battery + Psychosocial factor*Demographic characteristic  β_Psychosocial_ represents change in years of epigenetic age (or per-year acceleration of aging for DunedinPACE) for 1 SD increase in the mean psychosocial factor score; β_Demographic_ represents change in years of epigenetic age (or per-year acceleration of aging for DunedinPACE) for females/those with a college degree; β_Interaction_ represents difference in years of epigenetic age (or per-year acceleration of aging for DunedinPACE) for 1 SD increase in mean psychosocial factor score between levels of demographic variables.  We only tested for interactions when the main effects of both the exposure of interest (psychosocial stress, loneliness, depressive symptoms) and demographic factor were associated with EAA in Model 1.  P-value <0.05 is bolded. | | | | | | | | |

| Supplemental Table 8. Two-way interaction between psychosocial factors/depressive symptoms and educational attainment (no degree vs. HS degree) on epigenetic age acceleration (Model 1; N=2,681). | | | | | | | | |
| --- | --- | --- | --- | --- | --- | --- | --- | --- |
| **Interaction Term** | **Exposure** | **EAA measure** | **β_Psychosocial_** | **P_Psychosocial_** | **β_HSdegree_** | **P_HSdegree_** | **β_Interaction_** | **P_Interaction_** |
| *Exposure*HS Degree* | Psychosocial Stress | PhenoAge | 0.42 | 0.20 | -0.64 | 0.13 | 0.03 | 0.94 |
|  |  | GrimAge | 0.39 | 0.06 | **-1.16** | **7.42E-06** | 0.09 | 0.67 |
|  |  | DunedinPACE | 0.009 | 0.16 | **-0.03** | **4.08E-05** | 0.006 | 0.38 |
|  | Loneliness | PhenoAge | 0.26 | 0.47 | -0.62 | 0.14 | 0.27 | 0.48 |
|  |  | GrimAge | **0.49** | **0.03** | **-1.06** | **5.95E-05** | -0.29 | 0.23 |
|  |  | DunedinPACE | 0.005 | 0.51 | **-0.03** | **6.27E-05** | 0.006 | 0.43 |
|  | Depressive Symptoms | GrimAge | 0.33 | 0.07 | **-1.08** | **6.44E-05** | 0.11 | 0.61 |
|  |  | DunedinPACE | **0.014** | **0.02** | **-0.03** | **4.57E-04** | 0.005 | 0.43 |
| Interaction Model: Epigenetic age acceleration ~ Psychosocial factor + Sex + Educational attainment + Marital status + Employment + Has child + Top 10 genetic ancestry PCs + WBC proportion + Year of psychosocial battery + Psychosocial factor*Demographic characteristic  β_Psychosocial_ represents change in years of epigenetic age (or per-year acceleration of aging for DunedinPACE) for 1 SD increase in the mean psychosocial factor score; β_HSdegree_ represents change in years of epigenetic age (or per-year acceleration of aging for DunedinPACE) for those with a HS degree; β_Interaction_ represents difference in years of epigenetic age (or per-year acceleration of aging for DunedinPACE) for 1 SD increase in mean psychosocial factor score between individuals with and without a HS degree.  We only tested for interactions when the main effects of both the exposure of interest (psychosocial stress, loneliness, depressive symptoms) and educational attainment were associated with EAA in Model 1.  P-value <0.05 is bolded. | | | | | | | | |

| Supplemental Table 9. Total variability in epigenetic age acceleration explained by psychosocial factors (FDR q<0.05). | | | | |
| --- | --- | --- | --- | --- |
|  | **Percent Variance Explained (PVE)** | | | |
|  | **HannumAA** | **PhenoAA** | **GrimAA** | **DunedinPACE** |
| **Model 1 (N=2,681)** | | | | |
| Psychosocial Stress | 1.47% | 2.86% | 4.68% | 1.99% |
| Loneliness | 1.46% | 4.08% | 1.37% | 1.50% |
| **Model 2 (N=2,656)** | | | | |
| Psychosocial Stress | 0.83% | 0.93% | 0.13% | 0.07% |
| Loneliness | 0.90% | 2.35% | 0.13% | 0.36% |
| Model 1: Epigenetic age acceleration ~ Psychosocial determinant + Gender + Educational attainment + Marital Status + Employment + Has child + Top 10 genetic ancestry PCs + WBC proportion + Year of psychosocial battery  Model 2: Model 1 + Smoking status + Alcohol use + BMI | | | | |

| Supplemental Table 10. Mediation by depressive symptoms of the relationships between psychosocial stress/loneliness and epigenetic age acceleration (Model 2; N=2,656). | | | | | | | | | |
| --- | --- | --- | --- | --- | --- | --- | --- | --- | --- |
|  | HannumAA | | PhenoAA | | GrimAA^*^ | | DunedinPACE | | |
|  | β (95% CI) | P-value | Mediation Effect  (95% CI) | P-value | Mediation Effect  (95% CI) | P-value | Mediation Effect  (95% CI) | | P-value |
| **Psychosocial Stress** | | | | | | | | | |
| Total Effect | 0.23 (0.03, 0.43) | 0.02 | 0.29 (0.02, 0.57) | 0.03 | -- | -- | **0.007 (0.002, 0.012)** | | **0.01** |
| Direct Effect | 0.17 (-0.04, 0.38) | 0.11 | 0.30 (0.01, 0.59) | 0.04 | -- | -- | **0.003 (-0.002, 0.009)** | | **0.23** |
| Indirect Effect | 0.06 (-0.002, 0.12) | 0.06 | -0.01 (-0.10, 0.07) | 0.80 | -- | -- | **0.003 (0.002, 0.005)** | | **5.82E-05** |
| Proportion Mediated | 0.26 | -- | -0.04 | -- | -- | -- | **0.50** | | **--** |
| **Loneliness** | | | | | | | | | |
| Total Effect | 0.25 (0.06, 0.44) | 0.009 | 0.43 (0.17, 0.69) | 0.001 | -- | -- | **0.007 (0.002, 0.012)** | | **0.004** |
| Direct Effect | 0.21 (0.01, 0.41) | 0.04 | 0.44 (0.17, 0.72) | 0.002 | -- | -- | **0.005 (-0.0003, 0.01)** | | **0.06** |
| Indirect Effect | 0.04 (-0.02, 0.10) | 0.16 | -0.01 (-0.09, 0.06) | 0.67 | -- | -- | **0.002 (0.001, 0.004)** | | **7.45E-04** |
| Proportion Mediated | 0.16 | -- | -0.04 | -- | -- | -- | **0.33** | | **--** |
| Indirect effects with p-value <0.05 are bolded.  ^*^ Mediation was not assessed because the total effects of psychosocial factors and GrimAA were not significant (p-value>0.05). | | | | | | | |  |  |

| Supplemental Table 11. Mediation by depressive symptoms of the relationships between psychosocial stress/loneliness and DunedinPACE in females. | | | | | | |  |
| --- | --- | --- | --- | --- | --- | --- | --- |
|  | DunedinPACE | | | | | |  |
|  | **Model 1 (N=1,585)** | | | **Model 2 (N=1,565)** | | |  |
|  | Mediation Effect  (95% CI) | P-value | Statistical Power | Mediation Effect  (95% CI) | P-value | Statistical Power |  |
| **Psychosocial Stress** |  |  |  |  |  |  |  |
| Total Effect | **0.018 (0.010, 0.025)** | **6.05E-06** | 0.97 | **0.011 (0.004, 0.019)** | **0.002** | 0.90 |  |
| Direct Effect | **0.013 (0.005, 0.021)** | **0.002** |  | **0.008 (0.0005, 0.015)** | **0.04** |  |  |
| Indirect Effect | **0.005 (0.002, 0.007)** | **3.03E-04** |  | **0.004 (0.001, 0.006)** | **0.004** |  |  |
| Proportion Mediated | **0.28** | **--** |  | **0.31** | **--** |  |  |
| **Loneliness** |  |  |  |  |  |  |  |
| Total Effect | **0.012 (0.004, 0.019)** | **0.002** | 0.99 | **0.010 (0.003, 0.017)** | **0.004** | 0.98 |  |
| Direct Effect | **0.009 (0.001, 0.016)** | **0.02** |  | **0.008 (0.001, 0.015)** | **0.03** |  |  |
| Indirect Effect | **0.003 (0.0007, 0.005)** | **0.009** |  | **0.002 (0.0001, 0.004)** | **0.04** |  |  |
| Proportion Mediated | **0.25** | **--** |  | **0.20** | **--** |  |  |
| Indirect effects with p-value <0.05 are bolded.  Post-hoc statistical power was calculated using Monte Carlo Power Analysis for Indirect Effects. | | | | | | |  |
|  | |  |  |  |  |  |  |

| Supplemental Table 12. Mediation by depressive symptoms of the relationships between loneliness and epigenetic age acceleration in individuals without a college degree (Model 1; N=1,971). | | | | | | | | | | | | |
| --- | --- | --- | --- | --- | --- | --- | --- | --- | --- | --- | --- | --- |
|  |  | PhenoAA | | |  | GrimAA | | |  | DunedinPACE | | |
|  | Mediation Effect  (95% CI) | | P-value | Statistical Power | Mediation Effect  (95% CI) | | P-value | Statistical Power | Mediation Effect  (95% CI) | | P-value | Statistical Power |
| Total Effect | 0.61 (0.30, 0.92) | | 9.82E-05 |  | **0.28 (0.08, 0.48)** | | **0.005** |  | **0.012 (0.006, 0.018)** | | **2.37E-04** | 0.99 |
| Direct Effect | 0.63 (0.31, 0.95) | | 1.23E-04 | 0.26 | **0.18 (-0.02, 0.38)** | | **0.08** | 0.90 | **0.008 (0.002, 0.015)** | | **0.01** |  |
| Indirect Effect | -0.02 (-0.12, 0.09) | | 0.75 |  | **0.10 (0.04, 0.17)** | | **0.002** |  | **0.004 (0.002, 0.006)** | | **5.49E-04** |  |
| Proportion Mediated | -0.03 | | -- |  | **0.36** | | **--** |  | **0.31** | | **--** |  |
| Indirect effects with p-value <0.05 are bolded.  Post-hoc statistical power was calculated using Monte Carlo Power Analysis for Indirect Effects. | | | | | | | | | | | | |

| Supplemental Table 13. Mediation by depressive symptoms of the relationships between loneliness and epigenetic age acceleration after removing one item from CES-D measure. | | | | | | | | | |
| --- | --- | --- | --- | --- | --- | --- | --- | --- | --- |
|  | HannumAA | | PhenoAA | | GrimAA^*^ | | DunedinPACE | | |
|  | β (95% CI) | P-value | Mediation Effect  (95% CI) | P-value | Mediation Effect  (95% CI) | P-value | Mediation Effect  (95% CI) | | P-value |
| **Model 1 (N=2,681)** | | | | | | | | | |
| Total Effect | 0.32 (0.13, 0.51) | 8.70E-04 | 0.50 (0.24,0.76) | 1.73E-04 | **0.21 (0.05, 0.37)** | **0.009** | **0.010 (0.004, 0.015)** | | **3.14E-04** |
| Direct Effect | 0.28 (0.09, 0.48) | 0.004 | 0.50 (0.23, 0.77) | 2.86E-04 | **0.15 (-0.02, 0.31)** | **0.08** | **0.007 (0.001, 0.012)** | | **0.02** |
| Indirect Effect | 0.06 (-0.01, 0.09) | 0.13 | -0.0009 (-0.06,0.06) | 0.98 | **0.07 (0.03, 0.11)** | **0.001** | **0.003 (0.002, 0.005)** | | **8.72E-06** |
| Proportion Mediated | 0.12 | -- | -0.001 | -- | **0.31** | **--** | **0.32** | | **--** |
| **Model 2 (N=2,656)** | | | | | | | | | |
| Total Effect | 0.25 (0.06, 0.44) | 0.009 | 0.43 (0.17, 0.69) | 0.001 | **--** | **--** | **0.007 (0.002, 0.012)** | | 0.004 |
| Direct Effect | 0.22 (0.03, 0.42) | 0.03 | 0.44 (0.17, 0.72) | 0.001 | **--** | **--** | **0.005 (0.00001, 0.010)** | | **0.04** |
| Indirect Effect | 0.03 (-0.02, 0.07) | 0.25 | -0.01 (-0.08, 0.05) | 0.62 | **--** | **--** | **0.002 (0.0009, 0.003)** | | **5.14E-04** |
| Proportion Mediated | 0.11 | -- | -0.04 | -- | **--** | **--** | **0.30** | | **--** |
| Indirect effects with p-value <0.05 are bolded.  ^*^Mediation was not assessed in Model 2 because the total effect of loneliness and GrimAA was not significant (p-value > 0.05). | | | | | | | |  |  |
